# Analysis of the efficacy of drilling decompression autologous bone marrow and allogeneic bone grafting in the treatment of HIV-positive patients with early osteonecrosis of the femoral head

**DOI:** 10.1186/s12891-023-07039-9

**Published:** 2023-11-21

**Authors:** Shengtao Li, Jie Wang, Rui Ma, Changsong Zhao, Zhengrong Gao, Xuemin Quan, Qiang Zhang

**Affiliations:** 1 Department of Orthopedics, Beijing Ditan Hospital, Capital Medical University, No.8, Jingshun East Street, Chaoyang District, Beijing, 100015 China; 2 Department of Orthopedics, Beijing Chaoyang Hospital, Capital Medical University, No. 8, Workers Stadium South Road, Chaoyang District, Beijing, 100020 China

**Keywords:** Human immunodeficiency virus, Osteonecrosis of the femoral head, Drilling decompression, Autologous bone marrow, Allogeneic bone, Transplantation

## Abstract

**Objective:**

To investigate the efficacy of treating patients with HIV-positive osteonecrosis of the femoral head using drilled decompression autologous bone marrow and allogeneic bone grafting.

**Methods:**

40 patients (44 hips) with early osteonecrosis of the femoral head treated by drilling decompression autologous bone marrow and allogeneic bone grafting since October 2015 were retrospectively analyzed, among which 20 patients (24 hips) were HIV-positive patients with early osteonecrosis of the femoral head, 16 males and 4 females, age 22–43 years, average 39.6 ± 10.18 years, and 20 patients (20 hips) in the same period HIV-negative early osteonecrosis of the femoral head patients, 13 males and 7 females, aged 48–78 years, mean 63.50 ± 7.94 years were negative controls. General information including ARCO stage, Harris score, VAS score, hematological indexes including CD4^+^ T lymphocyte count, and HIV viral load was recorded for all patients before surgery. All patients were operated on by drilling and decompression of the necrotic area, harvesting autologous iliac bone marrow with allogeneic bone, and bone grafting through the decompression channel. The patients were followed up regularly at 6, 12, and 24 months after surgery and annually thereafter, and the repair of the necrotic femoral head was observed by reviewing the frontal and lateral X-ray, CT or MRI of the hip joint, and the complications and functional recovery of the hip joint was counted and compared between the two groups.

**Results:**

All patients were followed up, and the ARCO stages in the HIV-positive group were stage I 2 hips, stage IIA 6 hips, stage IIB 8 hips, stage IIC 6 hips, and stage III 2 hips, with a follow-up time of 12 to 60 months and a mean of 24.6 months. In the negative control group, there were 3 hips in ARCO stage I, 7 hips in stage IIA, 5 hips in stage IIB, 3 hips in stage IIC, and 2 hips in stage III, and the follow-up time ranged from 13 to 62 months, with an average of 24.8 months. The Harris score and VAS score of the hip in both groups improved significantly at 6 months postoperatively compared with those before surgery (P < 0.001). The difference between the Harris score of the hip in the positive group at 24 months postoperatively compared with that at 6 months postoperatively was statistically significant, but the VAS score at 24 months postoperatively compared with that at 6 months postoperatively was not statistically significant. In the negative group, there was no statistically significant difference in the Harris score and VAS score of the hip at 24 months postoperatively compared with those at 6 months postoperatively. In the positive group, there was a trend of continuous increase in hip BMD from the beginning of the postoperative period (P < 0.001). There was no statistically significant difference between the negative group and the positive group at the 24 months postoperatively follow-up except for the Harris score, which was statistically significant (P < 0.001), and the VAS score, which was statistically insignificant. At the 24 months postoperatively follow-up, patients in both groups had good recovery of hip function, and no complications such as vascular and nerve injury and fracture occurred during the perioperative period and follow-up period, and no complications related to incisional infection and pulmonary infection occurred during hospitalization.

**Conclusion:**

The treatment of early HIV-positive osteonecrosis of the femoral head patients with autologous bone marrow and allogeneic bone grafting by drilling and decompression to remove the tissue in the necrotic area of the femoral head can effectively stop the process of osteonecrosis of the femoral head and promoting femoral head repair in HIV-positive patients is a safe and effective method for treating HIV-positive patients with early osteonecrosis of the femoral head, and can effectively delay or postpone total hip replacement in patients.

## Introduction

With the use of highly active antiretroviral therapy (HAART), the survival of HIV-positive patients is significantly longer, and the number of patients requiring surgery for osteonecrosis of the femoral head (ONFH) is gradually increasing [1]. The etiology of HIV-related ONFH is not yet clear, but its incidence has been increasing since the introduction of HAART, especially in patients with protease inhibitors [2]. Studies have shown that HIV itself may be an independent risk factor, associated with adrenocorticosteroid use, hypercholesterolemia, hypertriglyceridemia, smoking, excessive alcohol consumption, antiphospholipid antibodies, and megestrol acetate [3]. The prevalence of ONFH in HIV-positive patients is statistically 10–100 times higher than in the HIV-negative population [4], and in addition, asymptomatic ONFH is common in HIV-positive patients, with 4.4% of asymptomatic HIV-positive patients showing signs of ONFH on MRI compared with 1.7% of HIV-negative patients [4, 5]. Drilling decompression autologous bone marrow and allograft bone grafting is an important method for the treatment of early ONFH, in which the necrotic femoral head is drilled to reduce intraosseous pressure, remove tissue from the necrotic area, and improve blood perfusion to the femoral head [6]. In the early phases, several surgical procedureshave been reported: core decompression,bone grafting coupled with mesenchymal stem cell injection [7].This study investigated the efficacy of drilled and decompressed autologous bone marrow and allogeneic bone grafts in the treatment of HIV-positive patients with osteonecrosis of the femoral head, as reported below.

## Materials and methods

### Patient inclusion and exclusion criteria

Inclusion criteria for the experimental group: ① HIV-positive patients diagnosed according to the AIDS Treatment Guidelines [8], with preoperative CD4^+^ T lymphocyte counts greater than 200/µL [9]; ② ONFH diagnosed according to the Chinese Clinical Guidelines for Adult Femoral Head Necrosis (2020) [10], with ARCO staging [11] confirmed by imaging as stage I, II and early stage III(III a) [12]; ③ none of the patients used drugs, or actively abused alcohol during the study period, with a strong intention to preserve the hip, agreed to adhere to the follow-up, and signed the informed consent before surgery. Exclusion criteria: patients with HIV-positive ONFH treated with total hip replacement (THR), patients with preoperative CD4^+^ T lymphocyte count less than 200/µL had elective surgery if not necessary [9]; patients who are in poor health due to high co-morbidities and for whom surgical treatment may be life-threatening; those with ONFH caused by trauma; those who did not sign informed consenters.

Inclusion criteria for the control group: HIV infection was excluded, and the rest was the same as the experimental group. Exclusion criteria: HIV-negative ONFH patients treated with THR, with ARCO stage III end-stage(III b) [12]or stage IV; patients who are in poor health due to high co-morbidities and for whom surgical treatment may be life-threatening; those with trauma-induced ONFH; and those who did not sign informed consent.

### General data

Since October 2015, 40 patients (44 hips) admitted to the Department of Orthopaedics, Beijing Ditan Hospital, Capital Medical University, for the treatment of early ONFH with drilled and decompressed autologous bone marrow and allogeneic bone grafting met the above inclusion criteria, of which 20 HIV-positive patients (24 hips) were in the experimental group, 2 hips in ARCO stage I, 6 hips in stage IIA, 8 hips in stage IIB, 6 hips in stage IIC, and 2 hips in early stage III. There were 16 males and 4 females, aged 22–43 years, with a mean of 39.60 ± 10.18 years. The follow-up time ranged from 12 to 60 months, with a mean of 24.6 months. The HIV-positive history ranged from 2.4 to 6.0 years, with a mean of 3.9 years, and all were treated with long-term oral tilapia antiviral regimens for 2 to 5 years since the diagnosis of HIV infection, with a mean of about 3 years.Preoperative CD4^+^ T lymphocyte counts ranged from 201 to 747 × 10^6^/L, with a mean of 428.25 ± 130.50/L; HIV RNA levels, i.e., viral load (VL), were undetectable to 647copies/ml, with a mean of < 50 copies/ml. the remaining 20 cases (20 hips) were negative controls, with ARCO stage I The remaining 20 cases (20 hips) were negative controls, with ARCO stage I 3 hips, stage IIA 7 hips, stage IIB 5 hips, stage IIC 3 hips, and stage III 2 hips. There were 13 males and 7 females, aged 48–78 years, with a mean of 63.50 ± 7.94 years. The follow-up time ranged from 13 to 62 months, with a mean of 24.8 months.

**Fig. 1:**
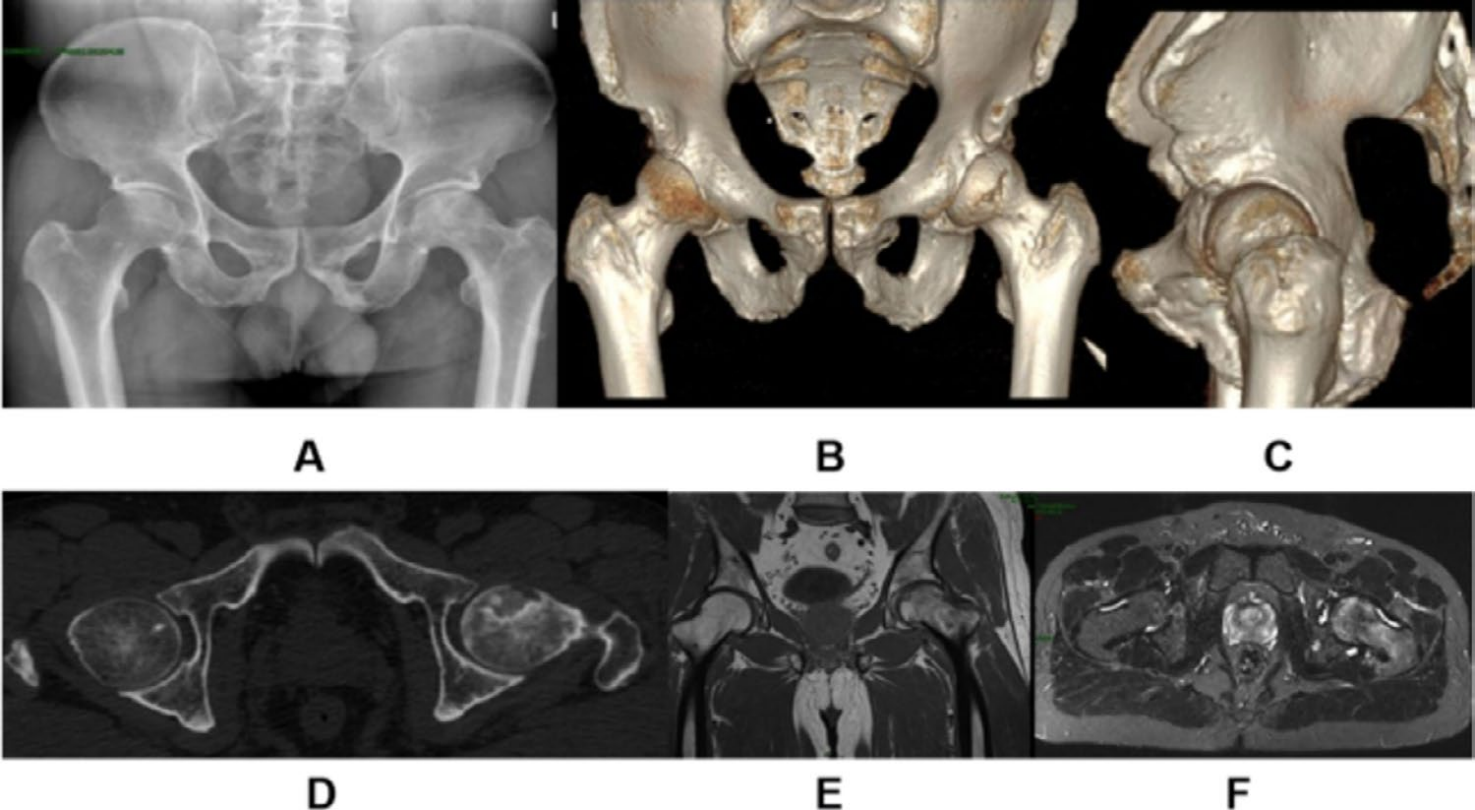
**(A~F)** Preoperative images of an HIV-positive ONFH patient (bilateral); bilateral hip pain for 3 months, aggravated by limited mobility and claudication for 1 month; bilateral hip ARCO stage IIc, treated with bilateral femoral head drilling and decompression combined with bone grafting and autologous bone marrow transplantation. Wear of the femoral head, narrowing of the joint space and uneven surface of the femoral head (X-ray A); significant necrotic area of the femoral head, wear and deformation, narrowing of the joint space and uneven surface of the femoral head (CT B,C,D); mixed signal within the femoral head, depression of the cartilage surface and narrowing of the joint space (MRI E,F)

According to the HIV infection classification system of the Centers for Disease Control and Prevention (CDC), which classifies the clinical stages of HIV infection [13], 4 cases in the experimental group were in clinical stage I, 16 cases in clinical stage II, and no patients in clinical stage III, with a history of ONFH of 1 to 5 years, with a mean of 2.9 years. Double hip ONFH patients were operated on in stages, with a surgical interval of 3 to 6 months, with a mean of 3.8 months. The general information of the two groups is shown in Table 1. Figure 1**(A~F)** shows the preoperative imaging presentation of an HIV-positive ONFH patient. This study was approved by the Ethics Committee of Beijing Ditan Hospital, Capital Medical University.

**Table 1:** General data and perioperative indicators of patients in both groups

Indicators	Group A (20 cases, 24 hips)	Group B (20 cases, 20 hips)	T-value	P-value
Age (years)	39.60 ± 10.18	63.50 ± 7.94	-7.07	0.019
Sex (male:female)	16∶4	13:7	-	-
Clinical stage of HIV infection (I:II:III)	4∶16∶0	-	-	-
ARCO stage (I:II:III)	2∶20∶2	3:15:2	-	-
CD4 + T-cell count (×106/L)	428.25 ± 130.50	-	-	-
CD8 + T-cell count (×106/L)	760.43 ± 254.69	-	-	-
CD4+/CD8 + ratio	0.49 ± 0.22	-	-	-
VL (copies/ml)	< 50	-	-	-
White blood cell count (×10^9^/l)	6.28 ± 1.55	5.68 ± 1.89	0.86	0.182
Neutrophil count (×10^9^/l)	4.19 ± 1.89	3.26 ± 1.50	1.41	0.372
Albumin (g/l)	48.12 ± 11.06	45.63 ± 3.54	0.62	0.218
Hemoglobin (g/l)	138.11 ± 20.48	150.40 ± 26.65	-1.22	0.918
Length of hospitalization (days)	16.17 ± 3.25	8.39 ± 1.65	5.62	0.027
Length of surgery (min)	120.71 ± 57.47	86.58 ± 24.27	1.52	0.009

*Note*: Group A - experimental group; Group B - control group

### Surgical methods

**Fig. 2:**
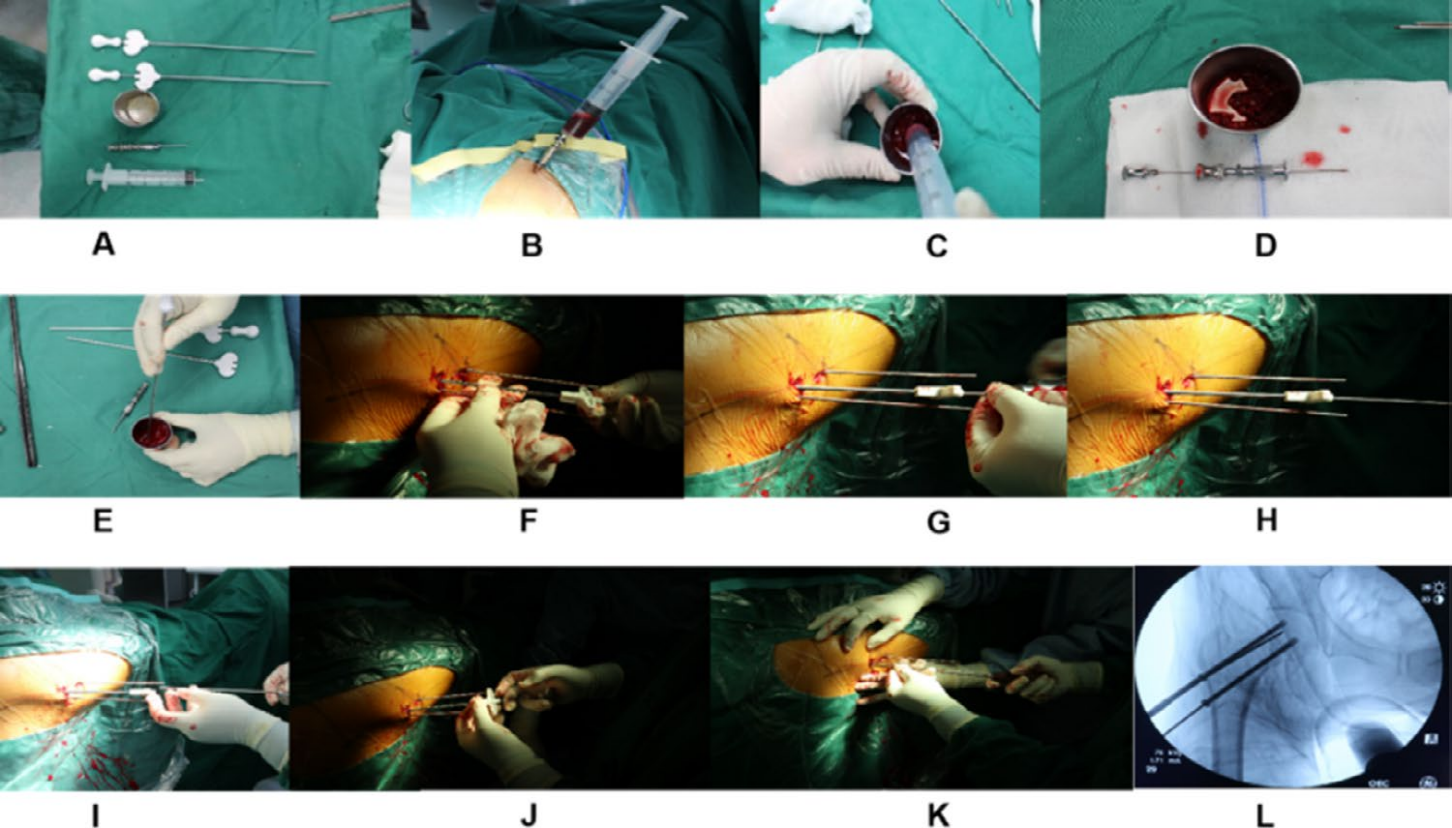
**(A~L)**Intraoperative situation The bone marrow is extracted by puncture at the anterior superior iliac spine, mixed with allograft bone, and the allograft bone mixed with bone marrow is implanted through a bone tunnel into the area of the necrotic cavity,and intraoperative fluoroscopy showed bone drilling through the decompression necrosis area

The surgery was performed by the same team of surgeons, and to avoid occupational exposure, the surgeons were required to wear protective equipment. The patient was placed supine on an orthopedic surgical traction bed with continuous epidural or general anesthesia, and the operating area was routinely disinfected and toweled. Intraoperative fluoroscopy was performed to verify the position of the guide needle, adjustment was made so that the guide needle reached the preoperative design position, and the 7.2 mm hollow drill was applied under fluoroscopy along with the guide needle to drill through the necrotic area, reaching 5 mm below the cartilage surface as appropriate, withdrawing the hollow drill, scraping the spoon to remove the necrotic area tissue, and the specimen was sent for pathological examination. The bone marrow was extracted from different directions with a 20 ml syringe using a bone puncture needle at the anterior superior iliac spine, and about 20–30 ml was aspirated, and mixed with allogeneic cancellous bone particles from Wuhan Union, sterilized by the instrument dealer (ethylene oxide, valid for 4 years) and set aside.Application of KYPHON V Premium Vertebroplasty System(Medtronic Sofamor Danek USA, Inc.),the bone marrow mud pusher in the instrument kit, pushes the allograft bone particles to implant and filling the decompression channel of the femoral head necrosis area and femoral neck, rams the bone graft particles through the tunnel under C-arm guidance, and after satisfactory fluoroscopy, the incision is sutured and dressed with a sterile dressing. The intraoperative situation is shown in Fig. 2**(A~L).**

### Perioperative treatment and efficacy evaluation

Pre-operative treatment: do safety and protection education for both doctors and patients, psychological preparation, pre-operative visit, preparation of articles, and preparation of protective gear for surgical personnel. VL was effectively controlled in HIV-positive patients who were admitted to the hospital and had their tilaï antiviral regimen replaced with a regimen containing an integrase inhibitor and maintenance of CD4^+^ T lymphocyte counts above 200/µL, with no significant adverse effects in the experimental group; aggressive management of comorbid underlying disease in elderly patients preoperatively. For hypertensive patients, preoperative adjustment of blood pressure was stabilized within 140/90 mmHg; for diabetic patients, perioperative glucose control with mentored insulin was performed with fasting or preprandial glucose 6.1–7.8 mmol/L and 2 h postprandial or random glucose 7.8–10.0 mmol/L; for patients with coronary artery disease, perfect cardiac enzymes, electrocardiogram, cardiac ultrasound, coronary CT angiography or coronary angiography, ask the anesthesiology and cardiology departments to assess tolerable surgery, discontinue antiplatelet drugs, and use low-molecular heparin calcium replacement therapy; for patients with hypoproteinemia, preoperative transfusion of human albumin ensured that patients’ serum albumin was greater than 30 g/L; Dual-energy X-ray absorptiometry (DXA) assessment of hip bone mineral density, for osteoporotic patients with DXA bone mineral density T values ≤ -2.5 SD, perioperative and post-discharge long-term oral triple antiosteoporosis drug treatment with alendronate, calcium, and vitamin D.

Special intraoperative treatment in the experimental group: the surgical staff wore protective equipment such as double gloves and eye protection devices, paid attention to not over-pursuing speed during surgical operations, paid attention to intraoperative protection such as strengthening isolation measures, using non-contact techniques for sharp delivery, suction hemostasis, needleless suturing, blunt needle suturing, etc.; AbbVita 160 was given intravenously to strengthen antiviral treatment to reduce the risk of postoperative complications and occupational exposure.

Postoperative treatment: Used disposable items such as masks and gowns were promptly placed in yellow plastic garbage bags, and instruments such as needles and blades were placed in the injurious waste receptacle and labeled “HIV-positive”. Spray 70% alcohol into the bag, seal it, and then incinerate it with a person in charge. The patient’s blood and secretions are put into containers with special signs and soaked in 70% alcohol for 1 h. The operating room equipment is coated with 2% glutaraldehyde, wiped with clean water, and then disinfected. The surgical staff is strictly disinfected after surgery, and once occupational exposure occurs, it is immediately reported to the relevant hospital departments and prophylactic medication is administered in the shortest possible time. Pay attention to the control of blood pressure, blood sugar, and albumin level after surgery, and instruct patients to move their lower limbs to prevent the formation of deep vein thrombosis in the lower limbs. According to individual conditions, partial weight-bearing with crutches for 6 weeks after surgery, gradually to full weight-bearing after 6 weeks, and complete de-crutching in 3 months. Patients in the experimental group continued and adhered to the original HAART protocol after surgery and discharge from the hospital.Long-term postoperative alendronate/zoledronate, vitamin D (1200 U/d) and calcium (1200 mg/d) for those with a clear diagnosis of osteoporosis. The perioperative-related indexes are shown in Table 1. Postoperative outpatient follow-up was performed at 6, 12, 24 months, and annually thereafter, and radiological assessments such as anterior flexion and frog leg lateral radiographs or MRI was performed at each follow-up visit. The values of the visual analog scale (VAS), Harris hip score, and hip bone mineral density (BMD) (measured by dual-energy X-ray) were recorded before surgery, 6 months after surgery, and 2 years after follow-up, as shown in Table 2.

**Table 2:** Changes in BMD values at different time points in the experimental group and comparison of Harris and VAS scores at different time points in the two groups

	Group A			Hip Harris score		T-value	P-value	VAS score					T-value	P-value
	BMD(g/cm^2^)	T-score	Z-score	Group A	Group B				Group A		Group B			
**Preoperative**	0.81 ± 0.12	−1.34 ± 0.69	−1.05 ± 0.67	55.57 ± 3.60	56.43 ± 3.10		−0.82	0.414	5.19 ± 0.98		5.05 ± 0.74		0.53	0.597
**6 months postoperatively**	0.91 ± 0.07	−0.67 ± 0.54	−0.46 ± 0.50	87.33 ± 2.47	88.05 ± 2.31		−0.96	0.340	1.24 ± 0.76		1.14 ± 0.72		0.41	0.682
**24 months postoperatively**	1.09 ± 0.06	0.47 ± 0.27	0.49 ± 0.37	85.05 ± 4.05	88.81 ± 2.08		−3.77	0.001	0.95 ± 0.74		1.05 ± 0.74		−0.41	0.679
** F-value**	58.117	71.179	52.319	556.31	1110.83		-	-	168.32		202.11		-	-
**P-value**	< 0.001	< 0.001	< 0.001	< 0.001	< 0.001		-	-	< 0.001		< 0.001		-	-

*Note*: Group A - experimental group; Group B - control group

### Statistical methods

SPSS 26.0 statistical software was used for analysis, and the measurement data were expressed as xˉ ± s. When the data were normally distributed, independent samples t-test was used for comparison between the baseline data of the two groups. One-way ANOVA was used to compare the follow-up scores between the two groups at different time points, and the LSD method was used for two-way comparisons. Independent samples t-test was used for comparison between the two groups. The Friedman or Kendall test was used when the data were not normally distributed. p < 0.05 was considered a statistically significant difference.

## Results

In the experimental group, the follow-up time ranged from 12 to 60 months, with a mean of 24.6 months and the hip Harris score improved from (55.57 ± 3.60) preoperatively to (87.33 ± 2.47) at 6 months postoperatively and (85.05 ± 4.05) at 24 months postoperatively follow-up, with a statistically significant difference (P < 0.001), and the VAS score decreased from (5.19 ± VAS scores decreased from preoperative (5.19 ± 0.98) to postoperative (1.24 ± 0.76) at 6 months postoperatively and to (0.95 ± 0.74) at the 24 months postoperatively follow-up, with a statistically significant difference between preoperative and postoperative VAS scores at 6 months postoperatively, but not between postoperative and 24 months postoperatively follow-up. At the 24 months postoperatively follow-up, the hip BMD improved significantly, and the difference was statistically significant when compared with preoperative and 6 months postoperatively (P < 0.001). In the control group, the follow-up time ranged from 13 to 62 months, with a mean of 24.8 months, and the hip Harris score improved from (56.43 ± 3.10) preoperatively to (88.05 ± 2.31) at 6 months postoperatively and (88.81 ± 2.08) at the 24 months postoperatively follow-up, with a statistically significant difference (P < 0.001) when comparing preoperatively and 6 months postoperatively. The difference was not statistically significant (P > 0.05) when comparing 6 months postoperatively to 24 months postoperatively, and there was no statistically significant difference (P > 0.05) when comparing the rest of the period with the experimental group, except for the statistically significant difference (P = 0.001) when comparing Harris scores at the 24months postoperatively follow-up. the VAS scores decreased from (5.05 ± 0.74) before surgery to (1.14 ± 0.72) at 6 months postoperatively, (1.05 ± 0.74) at the 24 months postoperatively follow-up, and the difference was statistically significant (P < 0.001) when comparing preoperative with 6 months postoperatively and no statistically significant (P > 0.05) when comparing 6 months postoperatively with 24 months postoperatively, and the difference in VAS scores was not statistically significant when comparing with all periods in the experimental group.

**Fig. 3:**
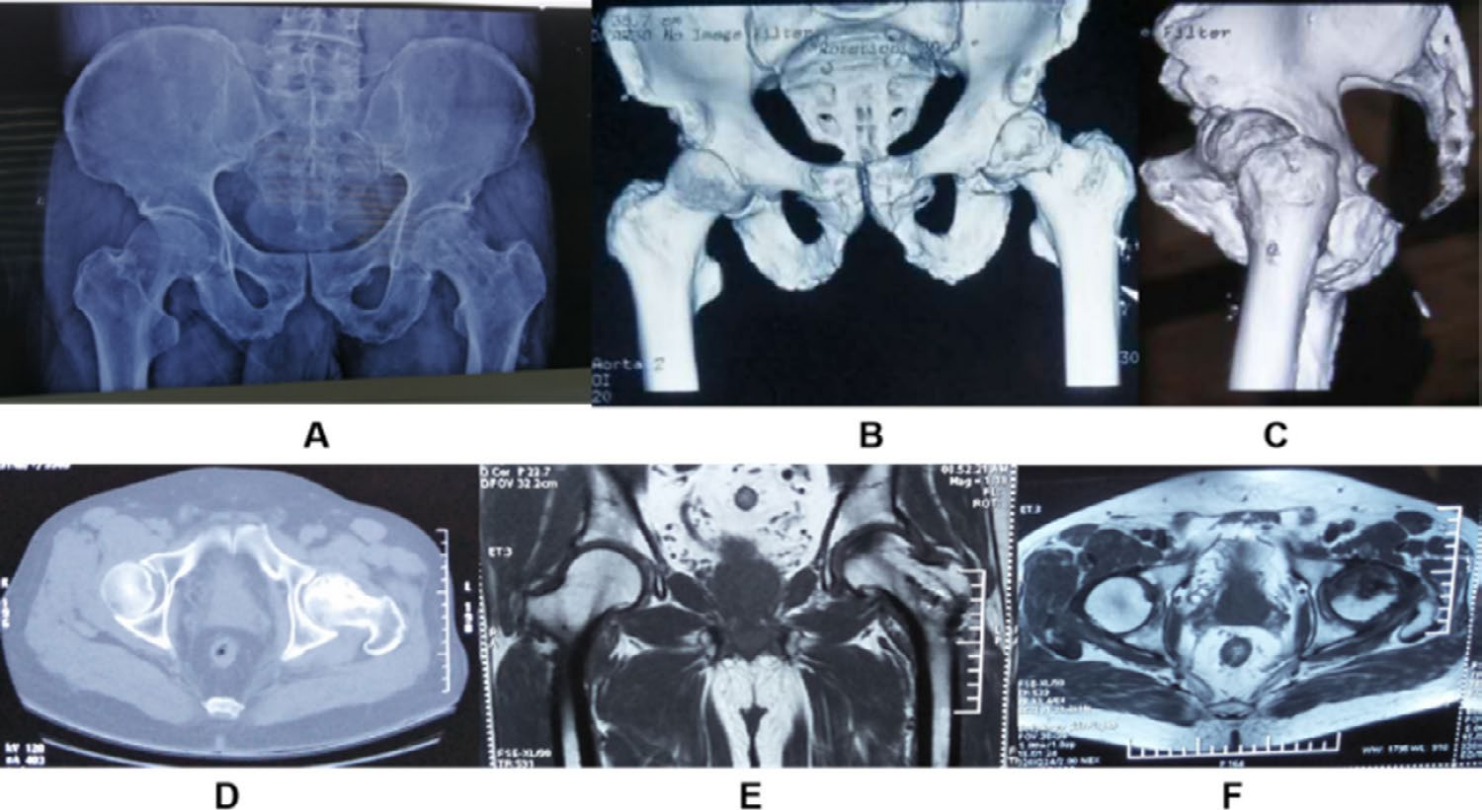
**(A~F)** Imaging performance at the 24 months postoperatively (bilateral) During follow-up, the patient did not see any significant narrowing and widening of the hip gaps bilaterally, and the X-ray showed bone filling in the capsular area. 2 years later, CT and MRI showed that the necrotic area was slowly replaced by some autologous bone, and the abnormal signal changes of bone and soft tissue were significantly restored compared with before, and the surface of the femoral head was less flat and did not collapse further, without pain and other There was no pain and other uncomfortable symptoms, and the patient was satisfied with the treatment:X-ray (A);CT (B,C,D);MRI (E,F)

There were no intraoperative complications such as vascular and nerve injuries and fractures, and no infection-related complications such as incisional infections and pulmonary infections during hospitalization; in the positive group,one case of oral cytomegalovirus infection and one case of Pneumocystis carinii pneumonia, which was cured after treatment; two cases of THR due to aggravation of ONFH on the operated side; three cases of pain due to weight-bearing on the operated side of the limb due to ONFH on the opposite side;And in the negative group, one case of the hip on the operated side due to external environmental, THR was performed in 3 cases due to aggravation of ONFH on the operated side during the follow-up period; 4 cases had pain due to weight bearing on the operated side due to ONFH on the contralateral side; 2 cases had THR on the operated side due to bone graft resorption and femoral head collapse 2 years after surgery. It is noteworthy that the trend of improvement in functional scores and imaging review within two years of follow-up was observed in both groups of patients who underwent borehole decompression osteotomy, while the final time of THR was about 3 to 5 years after 2 years of surgery and the cause of necrosis progression may be related to long-term oral hormone therapy and alcohol consumption. After evaluation, patients treated with THR reached preoperative ARCO stage IIIb or IV, with a preoperative Harris score of 50.71 ± 4.12 in such patients in the positive group and 49.26 ± 5.34 in the control group, with a significant improvement in functional scores after THR treatment.The complications of the patients during the postoperative and follow-up periods are shown in Table 3, and the relevant imaging performances during the follow-up period are shown in Fig. 3**(A~F)**.

**Table 3:** Complications during postoperative and follow-up periods in both groups

Complications	Group A(24 hips)	Group B(20 hips)	Statistic quantity	P-value
Opportunistic infections(n)				
Oral cytomegalovirus infection	1	0		
Pneumocystis carinii pneumonia	1	0		
Deep vein thrombosis(n)	0	0		
Hip pain on the operated side(n)	4	4		
THR for increased necrosis(n)	2	3	-	1.00
Resorption and collapse of implant THR on the operated side again(n)	1	2		
Contralateral ONFH(n)	3	4		
Total morbidity (%)	37.5	45	X^2^ = 0.25	0.42

*Note*: Group A - experimental group; Group B - control group

## Discussion

### Possible mechanisms of ONFH in HIV-positive patients

ONFH is a debilitating disease characterized by increased intraosseous pressure and reduced blood supply to the femoral head leading to progressive bone tissue necrosis, with unknown pathogenesis [14], in which trauma, hormones, and alcohol are the three main risk factors. In the case of HIV-positive patients, the pathogenesis of ONFH is related to the application of HAART regimens such as tenofovir disoproxil fumarate (TDF), protease inhibitors (PI) and the involvement of HIV itself in cytokine regulation, the effect on differentiation and apoptosis of mesenchymal stem cells (MSCs), osteoblasts, osteoclasts, etc. [15, 16], the development of bone metabolism disorders may be multifactorial, such as gender, age, weight, malnutrition, smoking, alcohol consumption, steroid hormones, and lipid metabolism disorders [17], and the combination of these risk factors with HIV infection and HAART side effects may be the ultimate cause of ONFH in HIV-positive patients. Studies have reported the incidence of ONFH in HIV-positive patients to be 0.4–4.4% [5, 18–20], with a mean age of 38.1–44.4 years, which corresponds to an ONFH incidence of only 0.010–0.135% in the general population, with an age of onset of 35–55 years [21, 22], with the advancement of medical technology and the increasing demand for minimally invasive and HIV positive patients are increasing, especially in young and middle-aged patients, it is especially urgent to find new operationally simple, safe and effective surgical methods to reduce occupational exposure of health care workers.

### Characteristics of ONFH in HIV-positive patients and special management in the perioperative period

HIV-positive ONFH patients have an uneven femoral head surface, most of the cartilage surface has folds, subchondral separation or fibrous tissue proliferation on the cartilage surface, yellowish uniform granular bone in the center of subchondral necrosis, cystic lesion formation, obvious sclerotic zone formation in the repair area, and multifocal granulation tissue proliferation. This group is more specific and requires special management measures in the perioperative period: preoperative HAART treatment is required for at least 1 month, VL is effectively controlled, the CD4^+^ T lymphocyte count was maintained above 200/µL, and no significant adverse effects were observed; most of the HIV-positive patients were younger than the negative patients (P = 019), and the preoperative indexes, such as inflammatory indexes, were not significantly different from those of the negative patients except for HIV-related indexes (Table 1), so the preoperative antiviral treatment was particularly important and had no significant adverse effect on the postoperative hip function. Therefore, preoperative antiviral treatment is particularly important and has no significant adverse effect on postoperative hip function recovery. HIV-positive patients have a significantly longer operative time than negative patients (P = 009), so special intraoperative protection measures are needed to reduce the risk of infection among healthcare workers. For postoperative management, HIV-positive patients also had a longer hospital stay than negative patients (P = 027), and postoperative attention was paid to the control of blood pressure, blood glucose, and albumin levels, and patients were instructed to move their lower limbs to prevent lower limb deep vein thrombosis. In this study, the patients with ONFH re-progression in the HIV-positive group may be related to HAART treatment, but in comparison, long-term adherence to HAART treatment is more beneficial.We introduced the concept of enhanced recovery after surgery (ERAS) in the treatment of HIV-positive patients, with emphasis on rapid viral load reduction, immunity enhancement, improvement of nutritional status, control of co-infections, prophylactic antibiotics, and anti-osteoporosis in the perioperative period.

### Surgical methods

Early surgical intervention can effectively delay the progression of necrosis and osteoarthritis [23], and early treatment before the advanced subchondral bone collapse in ONFH is essential to preserve the structure and function of the joint and prevent the need for THR (a common treatment option for advanced ONFH) [24]. Treatment of patients with early ONFH must address the following four issues to repair the necrotic area [25]: (1) improve blood flow in the femoral head and promote vascular regeneration; (2) effectively remove the necrotic bone; (3) reconstruct the cartilage in the collapsed area of the femoral head to restore its shape and improve the matching relationship between the femoral head and the acetabulum; and (4) improve the mechanical properties of the femoral head and prevent its collapse.

Drilling decompression can be used to manage small lesions in their early stages [26], as this procedure can effectively relieve the intraosseous pressure and venous stasis typical of ONFH [27, 28]. However, it alone may not be adequate in more extensive lesions, where insufficient activity of the osteoprogenitor cells does not provide sufficient bone reconstruction [29, 30].Notably, most of the presented RCTs performed the methods in patients with early-stage lesions and only two papers included patients with ARCO stage III lesions in their trials [31, 32], with variable results. Tabatabaee et al. [32] reported an imaging improvement in the two ARCO III patients treated with this surgical methods in their cohort, while Hauzeur and colleagues [31] did not see sufficient improvement in ARCO III patients undergoing the same treatment.And Migliorini F et al. for patients with femoral head osteonecrosis, the methods demonstrated reduced pain and lower rate of progression to total hip arthroplasty compared to core decompression as an isolated procedure [33]. At present, the combination of core decompression with stem cell transplantation (or concentrated autologous bone marrow single nucleus cell transplantation) is clinically effective in domestic medical institutions [34]. Combined stem cell transplantation has a better impact on ONFH outcomes compared to simple drill decompression,as also found by Rajagopal et al. [35]And in a meta-analysis,Sadile F et al. thought combining drill decompression with other techniques seems to provide better outcomes in ONFH. Detecting venous stasis and artery insufficiency could be the key to select the right indications for this kind of surgery and to reduce failures [36].

### Advantages, disadvantages, and efficacy of drilling and decompression of HIV femoral head necrosis with autologous bone marrow and allogeneic bone graft

Drilling decompression is important for HIV-positive early ONFH patients: on the one hand, the sclerotic bone is broken open through drilling to reach the necrotic area, so that the lesion area is fully decompressed; on the other hand, it can prevent the destruction of the femoral head cartilage, femoral head deformation and collapse. It works by using a 7.2 mm ring drill to percutaneously bore one or more tunnels from the greater trochanter, through the femoral neck, and into the subchondral bone of the femoral head [25, 37]. These tunnels reduce intraosseous pressure and may help restore blood flow to the femoral head, thus allowing for healing and preservation of the joint [38, 39]. In this study, combined autologous and allogeneic bone grafts were used to treat early ONFH by filling the bone defect after borehole decompression surgery with bone graft to provide a good carrier for MSC in bone marrow, maintain a high concentration of aggregates, and avoid local loss of MSC. Meanwhile, as a scaffold material, it can improve the local biomechanical strength, achieve the immediate reconstruction of the support of the necrotic area of the femoral head, provide a good scaffold for the formation of new bone, stop or delay the continued necrosis and collapse of the femoral head, and effectively solve the above four problems and good repair effect.

The long-term oral tenofovir-containing antiviral regimen of tenofovir in patients in the experimental group in this study [40, 41]. For HIV-positive patients who have been diagnosed with osteoporosis, we will prohibit the use of tenofovir-containing antiviral regimen upon admission and replace it with an integrase inhibitor-containing regimen in combination with anti-osteoporosis therapy, and patients in the experimental group in this study showed a continuous trend of increasing hip BMD during postoperative follow-up. With the development and marketing of new drugs, the current clinical trials of the antiviral regimens of Jefuccane, Pitocin, and injectable Epovetel have all confirmed safety and effectiveness with few adverse effects [42–44]. The use of the Jefukang regimen in orthopedic surgery has been shown to be superior to the tiramis regimen in terms of speed and efficiency of viral load reduction, speed and efficiency of recovery of CD4^+^ T lymphocyte counts, and postoperative complication rates as well [45]. It is possible to increase the use of the above-mentioned novel antiviral agents with low impact on BMD in the perioperative period and after discharge in patients with combined osteoporosis. The hip Harris score improved significantly in the HIV-positive group after surgery and at 6 months postoperatively compared to the HIV-positive group, and hip function also improved at 6 months postoperatively versus 24 months postoperatively follow-up, and VAS scores were significantly lower postoperatively compared with 6 months postoperatively, but the change was not significant at 6 months postoperatively versus 24months postoperatively follow-up. The Harris score and VAS score were not statistically significant compared with the negative control group at all periods, and the complications during follow-up were similar to those of the HIV-negative control group, during the follow-up period, the patient developed hip pain, which may be related to early weight-bearing and excessive exercise. However, the symptoms were significantly reduced compared with the preoperative period and the previous follow-up, and the imaging CT and MRI showed significant bone repair and improvement compared with the pre-treatment period, and the bone density gradually increased,suggesting that drilling decompression autologous bone marrow and allogeneic bone grafting for HIV-positive patients can achieve similar outcomes to those of the negative population.

## Limitions

The enrollment population of this study was relatively small with only 40 patients(44 hips), it was a single center study and all the surgeries were performed by the same group of surgeons, which may call into question the representativeness of the data and the validity of the results. Secondly this study has many limitations and may be affected by many different confounding factors such as follow-up time, improvement in surgical technique and postoperative follow-up only counting major complications, etc. We will customer service these shortcomings in subsequent studies such as expanding the sample size, collecting data from multiple centers, trying to control confounding factors, etc.

## Conclusion

The incidence of ONFH in HIV-positive patients is high, and most of them are young and middle-aged, in principle, the hip joint should be preserved as much as possible, and drilling decompression combined with autologous bone marrow transplantation can effectively reduce the symptoms of ONFH (even in stage III patients), and removing necrotic tissues of the femoral head by drilling can effectively stop the process of ONFH in HIV-positive patients and promote the repair of the femoral head, which is a safe and effective method of treating the patients with early stage of HIV-positive ONFH. It is a safe and effective method for treating patients with early stage HIV-positive ONFH, and can effectively delay or postpone THR in patients.

## Data Availability

All data generated or analyzed during this study are included in this article.

## List of abbreviations

HIV: Human Immunodeficiency Virus
HAART: highly active anti-retroviral therapy
ONFH: osteonecrosis of the femoral hea
THR: total hip replacement
BMD: bone mineral density

## References

[1] Vallabha T, Dhamangaonkar M, Sindgikar V (2017). Clinical Profile of Surgical Diseases with emergence of new problems in HIV + individuals. Indian J Surg.

[2] Permpalung N, Ungprasert P, Summachiwakij S (2014). Protease inhibitors and avascular necrosis: a systematic review and meta-analysis. Int J Antimicrob Agents.

[3] Mehta P, Nelson M, Brand A (2013). Avascular necrosis in HIV. Rheumatol Int.

[4] Morse CG, Mican JM, Jones EC (2007). The incidence and natural history of osteonecrosis in HIV-infected adults. Clin Infect Dis.

[5] Miller KD, Masur H, Jones EC (2002). High prevalence ofosteonecrosis of the femoral head in HIV–infected adults[J]. Ann Intern Med.

[6] Green KR, Hernandez-Jimenez JM, Isache CL (2018). Avascular necrosis: a growing concern for the HIV population. BMJ Case Rep.

[7] Quaranta M, Miranda L, Oliva F (2021). Osteotomies for avascular necrosis of the femoral head. Br Med Bull.

[8] Chinese Medical Association Infectious Diseases Branch AIDS Hepatitis C Group China Center for Disease Control and Prevention (2021). China AIDS Treatment Guidelines (2021 Edition). China AIDS STD.

[9] Surgical Group of the Academic Committee of the Chinese Association for STD and AIDS Prevention, Control, Surgical Group of the Chinese Society of Tropical Diseases and Parasitology, National Medical Center for Infectious Diseases (Beijing) (2021). Expert consensus on perioperative antiviral therapy for human immunodeficiency virus-infected patients in China (2nd edition). Chin J Experimental Clin Infect Dis (Electronic Version).

[10] Professional Committee on Bone Circulation and Osteonecrosis of the Orthopedic Physicians Branch of the Chinese Medical Association, Bone Microprosthetics Group of the Orthopedic Branch of the Chinese Medical Association., International Society of Bone Circulation China. Clinical guidelines for the treatment of adult femoral head necrosis in China (2020)[J]. Chin J Orthop 2020,40(20):1365–76.

[11] Yoon BH, Mont MA, Koo KH (2020). The 2019 Revised Version of Association Research Circulation Osseous Staging System of Osteonecrosis of the femoral head. J Arthroplasty.

[12] Hines JT, Jo WL, Cui Q (2021). Osteonecrosis of the femoral head: an updated review of ARCO on Pathogenesis, Staging and Treatment. J Korean Med Sci.

[13] Centers for Disease Control and Prevention (CDC) (2014). Revised surveillance case definition for HIV Infection–United States, 2014. MMWR Recomm Rep.

[14] Yu X, Zhang D, Chen X, Yang J (2018). Effectiveness of various hip preservation treatments for non-traumatic osteonecrosis of the femoral head: a network meta-analysis of randomized controlled trials. J Orthop Sci.

[15] Grigsby IF, Pham L, Gopalakrishnan R (2010). Downregulation of Gnas, Got2 and Snord32a following tenofovir exposure of primary osteoclasts[J]. Biochem Biophys Res Commun.

[16] Permpalung N, Ungprasert P, Summachiwakij S (2014). Protease inhibitors and avascular necrosis: a systematic review and meta–analysis[J]. Int J Antimicrob Agents.

[17] Zhao CS, Li X, Zhang Q (2015). El a1. Early Outcomes of Primary Total Hip Arthroplasty for osteonecrosis of the femoral head in patients with human immunodeficiency virus in China[J]. Chin Med J (Engl).

[18] Molia AC, Strady C, Rouger C (2004). Osteonecrosis in six HIV–infected patients receiving highly active antiretroviral therapy[J]. Ann Pharmacother.

[19] Gutiérrez F, Padilla S, Masiá M (2006). Osteonecrosis in patients infected with HIV: clinical epidemiology and natural history in a large case series from Spain[J]. J Acquir Immune Defic Syndr.

[20] Yombi JC, Vandercam B, Wilmes D (2009). Osteonecrosis of the femoral head in patients with type 1 human immunodeficiency virus Infection: clinical analysis and review[J]. Clin Rheumatol.

[21] McCurdie D, Roi DD, Sahu DA (2019). Severe bilateral knee osteonecrosis in a young man with human immunodeficiency virus[J]. Radiol Case Rep.

[22] Matos MA, Alencar RW, Matos SS (2007). Avascular necrosis of the femoral head in HIV infected patients[J]. Braz J Infect Dis.

[23] Fang T, Zhang EW, Sailes FC (2013). Vascularized fibular grafts in patients with avascular necrosis of femoral head: a systematic review and meta-analysis. Arch Orthop Trauma Surg.

[24] Xu Y, Jiang Y, Xia C (2020). Stem cell therapy for osteonecrosis of femoral head: opportunities and challenges. Regen Ther.

[25] Babis GC, Sakellariou V, Parvizi J (2011). Osteonecrosis of the femoral head. Orthopedics.

[26] Pierce TP, Jauregui JJ, Elmallah RK (2015). A current review of core decompression in the treatment of osteonecrosis of the femoral head. Curr Rev Musculoskelet Med.

[27] Ficat P, Arlet J, Vidal R (1971). Résultats thérapeutiques du forage-biopsie dans les ostéonécroses fémoro-caitales primitives (100 cas [Therapeutic results of drill biopsy in primary osteonecrosis of the femoral head (100 cases)]. Rev Rhum Mal Osteoartic.

[28] Hauzeur JP, Pasteels JL, Orloff S (1987). Bilateral non-traumatic aseptic osteonecrosis in the femoral head. An experimental study of incidence. J Bone Joint Surg Am.

[29] Gao YS, Zhang CQ (2010). Cytotherapy of osteonecrosis of the femoral head: a mini review. Int Orthop.

[30] Lieberman JR, Engstrom SM, Meneghini RM (2012). Which factors influence preservation of the osteonecrotic femoral head?. Clin Orthop Relat Res.

[31] Hauzeur JP, De Maertelaer V, Baudoux E (2018). Inefficacy of autologous bone marrow concentrate in stage three osteonecrosis: a randomized controlled double-blind trial. Int Orthop.

[32] Tabatabaee RM, Saberi S, Parvizi J (2015). Combining concentrated autologous bone marrow stem cells Injection with Core Decompression improves outcome for patients with early-stage osteonecrosis of the femoral head: a comparative study. J Arthroplasty.

[33] Migliorini F, Maffulli N, Eschweiler J (2021). Core decompression isolated or combined with bone marrow-derived cell therapies for femoral head osteonecrosis. Expert Opin Biol Ther.

[34] Zhao D, Cui D, Wang B (2012). Treatment of early stage osteonecrosis of the femoral head with autologous implantation of bone marrow-derived and cultured mesenchymal stem cells. Bone.

[35] Rajagopal M, Balch Samora J, Ellis TJ. Efficacy of core decompression as treatment for osteonecrosis of the hip: a systematic review. Hip Int 2012 Sep-Oct;22(5):489–93. 10.5301/HIP.2012.9748 23100153

[36] Sadile F, Bernasconi A, Russo S (2016). Core decompression versus other joint preserving treatments for osteonecrosis of the femoral head: a meta-analysis. Br Med Bull.

[37] Chughtai M, Piuzzi NS, Khlopas A (2017). An evidence-based guide to the treatment of osteonecrosis of the femoral head. Bone Joint J.

[38] Larson E, Jones LC, Goodman SB (2018). Early-stage osteonecrosis of the femoral head: where are we and where are we going in year 2018?. Int Orthop.

[39] Grassi M, Salari P, Massetti D (2020). Treatment of avascular osteonecrosis of femoral head by core decompression and platelet-rich plasma: a prospective not controlled study. Int Orthop.

[40] Han WM, Wattanachanya L, Apornpong T (2020). Bone mineral density changes among people living with HIV who have started with TDF-containing regimen: a five-year prospective study[J]. PLoS ONE.

[41] Baranek B, Wang S, Cheung AM (2020). The effect of tenofovir disoproxil fumarate on bone mineral density: a systematic review and meta-analysis[J]. Antivir Ther.

[42] Huang YS, Cheng CY, Liou BH (2021). Efficacy and Safety of Elvitegravir/Cobicistat/Emtricitabine/Tenofovir Alafenamide as Maintenance Treatment in HIV/HBV-Coinfected Patients[J]. J Acquir Immune Defic Syndr.

[43] Orkin C, DeJesus E, Sax PE (2020). Fixed-dose combination bictegravir, emtricitabine, and tenofovir alafenamide versus dolutegravir-containing regimens for initial treatment of HIV-1 Infection: week 144 results from two randomised, double-blind, multicentre, phase 3, non-inferiority trials[J]. Lancet HIV.

[44] Su B, Yao C, Zhao QX (2022). Long-acting HIV fusion inhibitor albuvirtide combined with ritonavir-boosted lopinavir for HIV-1-infected patients after failing the first-line antiretroviral therapy: 48-week randomized, controlled, phase 3 non-inferiority TALENT study[J]. J Infect.

[45] Ma R, Zhang Q, Zhang YS (2020). Preoperative rapid suppression of viral load by elvitegravir/cobicistat/emtricitabine/tenofovir alafenamide regimen in human immunodeficiency virus-positive fracture patients significantly reduces postoperative complications[J]. Chin Med J (Engl).

